# Preemptive action saves lives in a pandemic: closing the Grand Bazaar before the COVID-19 infection starts

**DOI:** 10.3906/sag-2008-169

**Published:** 2020-12-17

**Authors:** Abdullah Emre GÜNER, Kemal MEMİŞOĞLU, Şuayip BİRİNCİ

**Affiliations:** 1 Public Health Services, İstanbul Health Directorate, Ministry of Health of Turkey, İstanbul Turkey; 2 İstanbul Health Directorate, Ministry of Health of Turkey, İstanbul Turkey; 3 Ministry of Health of Turkey, İstanbul Turkey

Dear Editor,

We read with great interest the recent article entitled “The early impact of the Covid-19 pandemic on the global and Turkish economy” and we would like to share some of our views on the article. [1]

The COVID-19 pandemic began to take hold in December 2019 and has now affected almost the entire world. At the time of this writing, it has been recorded worldwide that there are more than 5 million cases and 300,000 deaths of COVID-19 (‘Coronavirus Update (Live)’, Worldometers, 2020). Treatment protocols and public health interventions have shown considerable variety amongst different countries, reflecting a general weakness in terms of evidence base for particular interventions. However, the most effective measures are usually guided by past experience in controlling outbreaks or pandemics. Such measures include physical distancing, isolation, and aggressive contact tracing.

We wish to add to the evidence base by describing an effective public health intervention taken to limit the spread of COVID-19: the decision taken to close the historic Grand Bazaar in Turkey, at the point where the effects of the pandemic were at their earliest stage in the country, just six days after the initial case of COVID-19 was recognized in the city.

Turkey as a whole initiated its own diagnosis, treatment and nationwide contact tracing protocols in early January 2020. When the first case of COVID-19 in Turkey was identified on 11th of March 2020, public health measures were swiftly put in place (‘Turkey confirms first case of coronavirus’, Anadolu Agency, 2020). Contact tracing was immediately initiated alongside case identification, and this effort brought up questions about closing of the Grand Bazaar. The Grand Bazaar is a five centuries-old, vast historic market that continues to draw large numbers of customers, including many international tourists, to its various shops. The market covers an area of 30,000 square meters. There are 4000 shops within it, providing employment for approximately 10,000 people. The population density is estimated to be around 0.3 persons per square meter. This corresponds to an interpersonal distance of less than 1.5 m, which is below the recommended social distance needed to prevent spread of infection (‘Coronavirus Disease 2019 (COVID-19)’, Centers for Disease Control, 2020).

The İstanbul Health Directorate responded to this health intelligence by sending a contact tracing team, consisting of 120 individuals to the Grand Bazaar to interview up to 10,000 individuals potentially working in the area. This major investigative effort was completed in one day under the authority of the Governor of Istanbul, who has the authority to decide to close the bazaar until adequate measures were taken.

On the day of investigation, 3912 people were screened for symptoms and assessed for contacts. Of the people screened, 136 (3.4%) were symptomatic. One hundred and eighty-nine Polymerase Chain Reaction (PCR) diagnostic tests for SARS-CoV-2 were undertaken, 38 (+20) of the tests were positive. These cases were treated according to the COVID-19 treatment algorithm developed by the Turkish Ministry of Health. The median age of PCR (+) cases was 38 (14–83), relatively young. The individuals diagnosed with COVID-19 had registered addresses corresponding to every district in İstanbul.

The rate of PCR positivity was relatively low amongst those screened, at 0.01 %. One case did; however, required careful consideration. The individual concerned had a history of overseas travel for 8 days prior to testing PCR+ for SARS-CoV-2. In the meantime, when he was likely already infected with SARS-CoV-2, this individual had been in contact with others at the Grand Bazaar. With this scenario in mind, the whole market was closed until the necessary measures were taken.

The subsequent lower-than-expected morbidity and mortality from COVID-19 within İstanbul (a city with a population of 15 million) shows that this decision was instrumental in tackling the disease. The regulatory decision took into consideration not solely the number of cases (low), but the conditions in which transmission is facilitated (i.e. high density of population and widespread distribution of potentially infected individuals). The followings are the key learnings from the İstanbul experience: regulatory responses need to be rapid and contact tracing needs to be exhaustive; where the exact mechanism of transmission is unclear, as with SARSCoV-2, assuming rapid spread is prudent; public health agencies need to work in contact with local and national government to ensure that appropriate decisions can bereached swiftly and appropriately on the basis of a detailed picture of any evolving outbreak to ensure that preemptive action can be taken.
